# Supplementary material for: Vitamin D Status Is Not Associated with Cognitive or Motor Function in Pre-School Ugandan Children
Source: Nutrients. 2020 Jun 3;12(6):1662. doi: 10.3390/nu12061662 (PMC7352320; doi:10.3390/nu12061662)
Supplement: Supplementary file 1 [file nutrients-12-01662-s001.pdf]

nutrients MDPI

SUPPLEMENTARY MATERIAL

Table S1: Comparison of baseline characteristics between children who were included in this analysis and children who were excluded due to loss to follow up or death or missing 25(OH)D and/ or cognitive and motor outcome data. 2

Table S2: Summary of cognitive and motor development scores. 3

Table S3: Univariable linear regression results for the association between participant characteristics and cognitive and motor outcomes. 4

Table S1: Comparison of baseline characteristics between children who were included in this analysis and children who were excluded due to loss to follow up or death or missing 25(OH)D and/ or cognitive and motor outcome data.

**Characteristics**	**Included participants (n=302)**	**Non-participants (n=2043)**	**P value***
Male sex (n,%)	146 (48.3)	550 (51.3)	0.36
Stunting at 5 years	101 (34.4)	193 (65.7)	0.003
Underweight at 5 years b	33 (11.1)	94 (9.9)	0.56
Wasting at 5 years c	14 (4.7)	74 (7.9)	0.07
Helminthic infections between birth and 5 years (n, %)	54 (17.9)	162 (15.2)	0.27
Asymptomatic malaria between birth and 5 years (n, %)	34 (11.3)	181 (16.9)	0.02
Malaria episodes between birth and 5 years (n, %)			
None	135 (44.7)	911 (47.2)	
1	65 (21.5)	406 (21.0)	
≥2	102 (33.8)	615 (31.8)	0.71
Maternal age (n,%)			
14-24	165 (54.6)	663 (61.9)	
25-34	110 (36.4)	365 (34.1)	
35+	27 (8.9)	44 (4.1)	0.001
Maternal education at enrollment to EMaBS			
Primary/ none	165 (54.8)	577 (53.9)	
Secondary	114 (37.9)	391 (36.5)	
Tertiary	22 (7.3)	102 (9.5)	0.49
Parity (n, %)			
1	54 (17.9)	301 (28.1)	
2-4	179 (59.3)	611 (57.0)	
5+	69 (22.9)	160 (14.9)	<0.001
Household socioeconomic status recorded at EMaBS enrollment g (n, %)			
1 (low)	16 (5.4)	50 (4.7)	
2	16 (5.4)	100 (9.5)	
3	82 (27.6)	336 (31.9)	
4	89 (29.9)	298 (28.3)	
5	69 (23.2)	216 (20.5)	
6 (high)	25 (8.4)	54 (5.1)	0.04
Location recorded at EMaBS enrolment (n, %)			
Urban	118 (39.1)	477 (45.1)	
Peri-urban	79 (26.2)	211 (19.9)	
Rural	105 (34.8)	370 (34.9)	0.06

*P value is from Pearson's chi-squared test

Table S2: Summary of cognitive and motor development scores.

Domain tested	Measure	N	Obtained Mean (SD) scores	Obtained scores (min, max)	Absolute scores (min, max)
Verbal and non-verbal IQ	Block design	333	8.4 (3.3)	0, 16	0, 16
	Picture vocabulary scale	336	17.4 (3.1)	6, 24	0, 24
Executive function	Verbal fluency	322	14.2 (7.8)	0, 36	a
	Picture search	336	4.1 (1.4)	0.3, 7.7	b
	Wisconsin card sort test	334	6.3 (3.9)	0, 12	0,12
Motor function	Coin box	330	9.9 (1.7)	3.5, 16.5	0, 20*
	Balancing on one leg	328	15.2 (11.3)	1.5, 57.5	0, 60*

SD, standard deviation; IQ, intelligence quotient; Min, minimum score; Max, maximum score. [a] One point is awarded for each correct name and a total score is calculated from the total correct names in a minute. [b] An average score is calculated from the total copies of pictures identified within 10 seconds.
*An average score is calculated after timed attempts of the tests.
Higher scores for the cognitive and motor tests indicate better performance.

Table S3: Univariable linear regression results for the association between participant characteristics and cognitive and motor outcomes.

Characteristics	Verbal and non-verbal IQ		Executive function		Motor function	
	β (95% CI)	P value	β (95% CI)	P value	β (95% CI)	P value
**Child characteristics**						
Age at 25(OH)D measurement (years) (n=302)						
1	0.01 (-0.84, 0.85)		-0.38 (-1.19, 0.44)		-0.43 (-1.14, 0.28)	
2	Reference		Reference		Reference	
3	-0.17 (-0.55, 0.21)		-0.23 (-0.59, 0.13)		-0.18 (-0.49, 0.13)	
4	-0.36 (-0.93, 0.22)	0.17*	0.02 (-0.53, 0.58)	0.79*	-0.07 (-0.55, 0.41)	0.71*
Sex (n=302)						
Male	Reference		Reference		Reference	
Female	-0.19 (-0.49, 0.10)	0.19	0.34 (0.05, 0.63)	0.02	0.32 (0.07, 0.57)	0.01
Height-for-age Z-scores (n=294)						
Normal (>-2 SD)	Reference		Reference		Reference	
Stunted (<-2 SD)	-0.44(-0.75, -0.12)	0.01	-0.15 (-0.46, 0.16)	0.35	-0.08 (-0.35, 0.19)	0.56
Weight-for-age Z-scores (n=297)						
Normal (>-2 SD)	Reference		Reference		Reference	
Underweight (<-2 SD)	-0.29 (-0.77, 0.19)	0.23	-0.14 (-0.61, 0.33)	0.56	-0.03 (-0.43, 0.38)	0.89
Weight-for- height Z-scores (n=297)						
Normal (>-2 SD)	Reference		Reference		Reference	
Wasted (<-2 SD)	0.17, (-0.54, 0.88)	0.64	-0.20 (-0.89, 0.49)	0.57	-0.01 (-0.59, 0.61)	0.99
Helminthic infection (n=302)						
Negative	Reference		Reference		Reference	
Positive	-0.10 (-0.49, 0.30)	0.63	-0.15 (-0.53, 0.23)	0.43	-0.27 (-0.60, 0.06)	0.11
Asymptomatic malaria parasitemia (n=302)						
Negative	Reference		Reference		Reference	
Positive	-0.23 (-0.71, 0.24)	0.34	-0.37 (-0.83, 0.09)	0.12	-0.05 (-0.45, 0.35)	0.79
Malaria episodes (n=302)						
None	Reference		Reference		Reference	
1	-0.14 (-0.53, 0.25)		-0.05 (-0.43, 0.34)		0.01 (-0.32, 0.34)	

Characteristics	Verbal and non-verbal IQ		Executive function		Motor function	
	β (95% CI)	P value	β (95% CI)	P value	β (95% CI)	P value
≥2	-0.30 (-0.64, 0.4)	0.08*	-0.08 (-0.42, 0.25)	0.62*	-0.03 (-0.32, 0.26)	0.86*
[1]Haemoglobin (n=286)						
Normal	Reference		Reference		Reference	
Anaemia	-0.22 (-0.54, 0.10)	0.18	-0.15 (-0.46, 0.16)	0.34	0.04 (-0.24, 0.31)	0.78
[2]Iron status (n=288)						
Normal	Reference		Reference		Reference	
Iron deficient	0.31 (-0.04, 0.65)	0.08	-0.06 (-0.39, 0.28)	0.73	0.20 (-0.09 0.49)	0.18
[3]Inflammation (n=299)						
No	Reference		Reference		Reference	
Yes	-0.13 (-0.49, 0.23)	0.48	-0.23 (-0.57, 0.12)	0.20	-0.11 (-0.41, 0.18)	0.45
[4] Child Albendazole (ABZ) treatment (n=302)						
ABZ	Reference		Reference		Reference	
Placebo	0.13 (-0.17, 0.43)	0.39	-0.06 (-0.36, 0.23)	0.67	-0.31 (-0.56,-0.06)	0.01
**Maternal characteristics**						
Age (years) (n=302)						
14-24	Reference		Reference			
25-34	-0.05 (-0.37, 0.27)		0.22 (-0.09, 0.53)		-0.16 (-0.43, 0.11)	
35+	0.13 (-0.41, 0.67)	0.87*	-0.39 (-0.91, 0.13)	0.78*	-0.28 (-0.73, 0.17)	0.13*
Education (n=301)						
Primary/none	-0.68 (-1.27, -0.10)		-0.39 (-0.96, 0.18)		0.17 (-0.33, 0.67)	
Secondary	-0.23 (-0.82, 0.37)		-0.12 (-0.71, 0.47)		0.22 (-0.29, 0.73)	
Tertiary	Reference	0.001*	Reference	0.05*	Reference	0.82*
Parity (n=302)						
1	Reference		Reference		Reference	
2-4	-0.07 (-0.48, 0.33)		-0.18 (-0.57, 0.21)		0.04 (-0.30, 0.38)	
5+	-0.19 (-0.67, 0.28)	0.40*	-0.28 (-0.84, 0.08)	0.10*	-0.14 (-0.54, 0.26)	0.43*
[4] ABZ treatment in pregnancy (n=302)						
ABZ	Reference		Reference		Reference	
Placebo	-0.11 (-0.41, 0.19)	0.46	-0.07 (-0.36, 0.22)	0.64	0.02 (-0.23, 0.28)	0.85
[4] Praziquantel treatment in pregnancy (n=302)						

Characteristics	Verbal and non-verbal IQ		Executive function		Motor function	
	β (95% CI)	P value	β (95% CI)	P value	β (95% CI)	P value
Praziquantel	Reference		Reference		Reference	
Placebo	0.14 (-0.16, 0.44)	0.35	-0.07 (-0.36, 0.23)	0.66	0.17 (-0.08, 0.42)	0.18
Household socioeconomic status (n=297)						
1(Lowest)	-0.63 (-1.46, 0.19)		-0.16 (-0.96, 0.65)		0.38 (-0.32, 1.08)	
2	-0.47 (-1.29, 0.35)		-0.48 (-1.28, 0.33)		-0.12 (-0.82, 0.58)	
3	-0.29 (-0.87, 0.30)		-0.23 (-0.81, 0.34)		0.39 (-0.11, 0.89)	
4	-0.23 (-0.82, 0.35)		-0.20 (-0.77, 0.37)		0.45 (-0.04, 0.95)	
5	0.34 (-0.26, 0.94)		0.19 (-0.39, 0.78)		0.41 (-0.10, 0.92)	
6 (Highest)	Reference	0.002*	Reference	0.06*	Reference	0.89*
Household Location (n=302)						
Urban	Reference		Reference		Reference	
Peri-urban	-0.16 (-0.54, 0.21)		-0.47 (-0.83, -0.10)		-0.15 (-0.47, 0.17)	
Rural	-0.18 (-0.53, 0.17)	0.29*	-0.35 (-0.68, -0.01)	0.04*	0.002 (-0.29, 0.29	0.99*

CI, confidence interval; SD, standard deviation. [1]Anaemia was defined as haemoglobin <11g/dL; [2]iron deficiency as ferritin <12 μg/L; [3]inflammation as C-reactive protein >5 mg/L; [4]anthelminthic treatment in primary study. *P value for linear trend.
